# Supplementary material for: A mechanical model of ocular bulb vibrations and implications for acoustic tonometry
Source: PLoS One. 2024 Jan 18;19(1):e0294825. doi: 10.1371/journal.pone.0294825 (PMC10796012; doi:10.1371/journal.pone.0294825)
Supplement: S1 File — (PDF) [file pone.0294825.s001.pdf]

Supplementary material to the paper: A mechanical model of ocular bulb vibrations and implications for acoustic tonometry

Nicoletta Tambroni,[1] Giuseppe Tomassetti,[2*] Silvia Lombardi[3] and Rodolfo Repetto[1]

[1]Department of Civil, Chemical and Enivironmental Engineering, University of Genoa, Genoa, Italy

[2] Dipartimento di Ingegneria, Industriale, Elettronica e Meccanica, Rome 3 University, Rome, Italy

[3]Gran Sasso Science Institute, L'Aquila, Italy

[*]Corresponding author

This supplementary document describes in detail the derivation of the characteristic equations that determine the eigenvalues of the system, computed in the main paper.

Motion equations

We model the eye as a composite structure, consisting of a viscoelastic material, the vitreous humor, which occupies a spherical domain B_R of radius R centered at the origin, enclosed within a thin elastic shell, representing the corneo-scleral complex. We consider an equilibrium state whereby the vitreous humor is at a pressure p, sustained by a state of stress in the corneo-scleral shell, and we study small-amplitude vibrations about this state.

The equations that govern the evolution of the displacement $\boldsymbol{u}$ in the vitreous humor are

$$\left.\begin{aligned} &\rho_{\rm v}\ddot{\boldsymbol{u}} = {\rm div}\boldsymbol{S}, \\ &{\rm div}\boldsymbol{u} = 0, \end{aligned}\right. \qquad \text{in } B_R \tag{1}$$

where $\rho_{\rm v}$ is the mass density of the vitreous humor, a superposed dot denotes partial differentiation with respect to time, and $\boldsymbol{S}$ is the increment of the Piola (nominal) stress with respect to the reference state. For the incremental stress we adopt the following constitutive equations:

$$\boldsymbol{S} = p\nabla\boldsymbol{u}^\top + \boldsymbol{\Sigma}, \qquad \boldsymbol{\Sigma}(x,t) = -q(x,t)\boldsymbol{I} + 2\int_{-\infty}^{t} G(t-s)\boldsymbol{D}(x,\dot{\mathbf{u}}(s))\mathrm{d}s, \tag{2}$$

where q is the pressure increment, and $\boldsymbol{D}(\dot{\mathbf{u}}) = \mathrm{sym}\,\mathrm{dev}\,\nabla\dot{\mathbf{u}}$ is the symmetric deviatoric part of the velocity gradient.

In the first of (2), the second term on the right-hand side accounts for the change of the Piola stress due to the initial pressure. This term, whose derivation may be found in [2], does not make any contribution to the bulk equations, since $\mathrm{div}\nabla\boldsymbol{u}^\top = \nabla\mathrm{div}\dot{\boldsymbol{u}} = 0$; however, it makes a contribution to the boundary conditions, as shown below. The relaxation function $G(t)$ describes the mechanical behavior of the material in the linear setting. For an elastic material, the relaxation function is proportional to the Heaviside function. For a viscous material, $G(t)$ is the Dirac delta centered at the origin.

We assume that there is no slip between the vitreous humor and the sclera. Accordingly, we set the displacement field on the sclera to be equal to the vector field $\boldsymbol{u}$ on the sphere $S_R = \partial B_R$. For convenience, we decompose $\boldsymbol{u}$ into its normal and tangential components, respectively, w and $\mathbf{v}$. Accordingly, on denoting by $\boldsymbol{n}$ the unit normal to S_R, we write

$$\boldsymbol{u} = w\boldsymbol{n} + \mathbf{v}, \qquad \mathbf{v}\cdot\boldsymbol{n} = 0, \qquad \text{on } S_R. \tag{3}$$

The equations that govern small displacements of the shell from its reference configuration have been derived in [5], and are given by

$$\left.\begin{aligned} \rho_s h\ddot{w} &= \mathrm{div_s}(\mathbf{P}\,\mathrm{div_s}\,\mathbf{M}) - \frac{1}{R}\mathbf{P}\cdot\mathbf{N} + \frac{p}{2}R\Delta_s w - \frac{p}{2}\mathrm{div_s}\mathbf{v} + \boldsymbol{n}\cdot\boldsymbol{b},\\ \rho_s h\ddot{\mathbf{v}} &= \mathbf{P}\,\mathrm{div_s}\,\mathbf{N} + \frac{1}{R}\mathbf{P}(\mathrm{div_s}\,\mathbf{M}) + \mathbf{P}\boldsymbol{b}, \end{aligned}\right. \quad \text{on } S_R. \tag{4}$$

Here ρ_s is the density of the sclera, h is its thickness, $\mathbf{P} = \boldsymbol{I} - \boldsymbol{n}\otimes\boldsymbol{n}$ is the projector on the plane orthogonal to $\boldsymbol{n}$, and

$$\boldsymbol{b} = -\boldsymbol{S}\boldsymbol{n}, \tag{5}$$

is the increment of the nominal traction (i.e., the traction per unit reference area) acting on the shell. By (4) and (5) we have

$$\left.\begin{aligned} \rho_s h\ddot{w} &= \mathrm{div_s}(\mathbf{P}\,\mathrm{div_s}\,\mathbf{M}) - \frac{1}{R}\mathbf{P}\cdot\mathbf{N} + \frac{p}{2}R\Delta_s w - \frac{p}{2}\mathrm{div_s}\mathbf{v} - \boldsymbol{n}\cdot\boldsymbol{S}\boldsymbol{n},\\ \rho_s h\ddot{\mathbf{v}} &= \mathbf{P}\,\mathrm{div_s}\,\mathbf{N} + \frac{1}{R}\mathbf{P}(\mathrm{div_s}\,\mathbf{M}) - \mathbf{P}\boldsymbol{S}\boldsymbol{n}, \end{aligned}\right. \quad \text{on } S_R. \tag{6}$$

The tensor fields $\mathbf{N}$ and $\mathbf{M}$ represent the increment of the nominal membrane-force and bending-moment tensors, and they are given by the constitutive equations

$$\begin{aligned} \mathbf{N} &= \frac{pR}{2}\nabla_s\boldsymbol{u} + h\left(2\mu\boldsymbol{\varepsilon} + \widetilde{\lambda}(\mathrm{tr}\,\boldsymbol{\varepsilon})\mathbf{P}\right) + \frac{1}{R}\mathbf{M},\\ \mathbf{M} &= \frac{h^3}{12}\left(2\mu\boldsymbol{\kappa} + \widetilde{\lambda}(\mathrm{tr}\,\boldsymbol{\kappa})\mathbf{P}\right), \end{aligned} \tag{7}$$

where μ and $\widetilde{\lambda}$ are suitable material moduli, which depend on the nature of the material comprising the shell, and

$$\begin{aligned} \boldsymbol{\varepsilon} &= \frac{1}{2}\left(\mathbf{P}\nabla_s\mathbf{v} + \nabla_s\mathbf{v}^\top\mathbf{P}\right) + \frac{w}{R}\mathbf{P},\\ \boldsymbol{\kappa} &= -\mathbf{P}\nabla_s\nabla_s w + \frac{1}{R}\mathbf{P}\nabla_s\mathbf{v} + \frac{1}{R}\nabla_s\mathbf{v}^\top\mathbf{P} + \frac{w}{R^2}\mathbf{P}, \end{aligned} \tag{8}$$

respectively, the stretching and bending strains. The symbol $\nabla_{\rm s}$ denotes the surface gradient on S_R. The surface divergence of the tangential vector field $\mathbf{v}$ is defined as $\mathrm{div}_s\mathbf{v} = \mathrm{tr}(\nabla_{\rm s}\mathbf{v}) = \mathbf{P} : \nabla_{\rm s}\mathbf{v}$, where the symbol : denotes contraction between tensors, while the surface divergence of $\mathbf{N}$ is defined by requiring that $\boldsymbol{a}\cdot\mathrm{div}_{\rm s}\,\mathbf{N} = \mathrm{div}_{\rm s}(\mathbf{N}^\top\boldsymbol{a})$ for every constant vector field $\boldsymbol{a}$ (note that since $\mathbf{N}\boldsymbol{n} = \mathbf{0}$, $\mathbf{N}^\top\boldsymbol{a}$ is a tangential vector field). The same definition holds for $\mathrm{div}_{\rm s}\mathbf{M}$.

It is worth remarking that the first term on the right-hand side of (7) and the first term on the right-hand side of (2) share the same nature: they take into account increments of the nominal stress that originate from the reference state being pre-stressed.

We next elaborate the last term in each of the motion equations (6). First we compute

$$\nabla\boldsymbol{u}^\top\boldsymbol{n} = \nabla(\boldsymbol{u}\cdot\boldsymbol{n}) - \nabla\boldsymbol{n}\boldsymbol{u} = \nabla w - \frac{1}{R}\mathbf{v}, \tag{9}$$

since $\nabla\boldsymbol{n} = \frac{1}{R}\mathbf{P}$. Thus,

$$\begin{aligned} &\boldsymbol{n}\cdot\nabla\boldsymbol{u}^\top\boldsymbol{n} = \nabla w\cdot\boldsymbol{n}, \\ &\mathbf{P}\nabla\boldsymbol{u}^\top\boldsymbol{n} = \nabla_s w - \frac{1}{R}\mathbf{v}, \end{aligned} \tag{10}$$

where $\nabla_s w$ is the superficial gradient of w. Accordingly, we have from (2)

$$\begin{aligned} &-\boldsymbol{n}\cdot\boldsymbol{S}\boldsymbol{n} = -p\nabla w\cdot\boldsymbol{n} - \boldsymbol{n}\cdot\boldsymbol{\Sigma}\boldsymbol{n}, \\ &-\mathbf{P}\boldsymbol{S}\boldsymbol{n} = p\Big(-\nabla_{\rm s}w + \frac{\mathbf{v}}{R}\Big) - \mathbf{P}\boldsymbol{\Sigma}\boldsymbol{n}. \end{aligned} \tag{11}$$

Then, the motion equations (6) can be rewritten as

$$\left.\begin{aligned} &\boldsymbol{n}\cdot\boldsymbol{\Sigma}\boldsymbol{n} + p\nabla w\cdot\boldsymbol{n} = \mathrm{div}_{\rm s}(\mathbf{P}\,\mathrm{div}_{\rm s}\,\mathbf{M}) - \frac{1}{R}\mathbf{P}\cdot\mathbf{N} + \frac{p}{2}R\Delta_s w - \frac{p}{2}\mathrm{div}_{\rm s}\mathbf{v} - \rho_s h\ddot{w}, \\ &\mathbf{P}\boldsymbol{\Sigma}\boldsymbol{n} = \mathbf{P}\,\mathrm{div}_{\rm s}\,\mathbf{N} + \frac{1}{R}\mathbf{P}(\mathrm{div}_{\rm s}\,\mathbf{M}) - p\nabla_s w + \frac{p}{R}\mathbf{v} - \rho_s h\ddot{\mathbf{v}}, \end{aligned}\right\} \text{ on } S_R. \tag{12}$$

Harmonic solutions

Frequency domain. We look for solutions of (1), (2), of the form

$$\boldsymbol{u}(x,t) = \mathrm{Re}(e^{\zeta t}\mathsf{u}(x,\zeta)), \qquad q(x,t) = \mathrm{Re}(e^{\zeta t}\mathsf{q}(x,\zeta)). \tag{13}$$

For any such solution, we have $\boldsymbol{S}(x,t) = \mathrm{Re}(e^{\zeta t}\mathsf{S}(x,\zeta))$, where $\mathsf{S} = p\nabla\mathsf{u}^\top + \mathbf{\Sigma}$, with

$$\mathbf{\Sigma}(x,\zeta) = -\mathsf{q}(x,\zeta)\boldsymbol{I} + \mathsf{G}(\zeta)(\nabla\mathsf{u}(x,\zeta) + \nabla^\top\mathsf{u}(x,\zeta)), \tag{14}$$

and

$$\mathsf{G}(\zeta) = \zeta\int_0^\infty e^{-\zeta\tau}G(\tau)\mathrm{d}\tau. \tag{15}$$

Thus, system (1) takes the form:

$$\left.\begin{aligned} &-\mathsf{G}(\zeta)\Delta\mathsf{u} + \nabla\mathsf{q} = -\zeta^2\rho_{\rm v}\mathsf{u} \\ &\mathrm{div}\,\mathsf{u} = 0. \end{aligned}\right. \quad \text{in } B_R. \tag{16}$$

By substituting (7), (8), and (11) into (12), keeping (13) in mind:

$$\begin{aligned}
\boldsymbol{n}\cdot\boldsymbol{\Sigma}\boldsymbol{n}+p\nabla\mathsf{w}\cdot\boldsymbol{n} &= \frac{1}{12}\Big(\frac{h}{R}\Big)^3\Big((2\mu+\widetilde{\lambda})\left(-R^3\Delta_s\Delta_s\mathsf{w}\right)\\
&\qquad + 2\mu R^2\,\mathrm{div}_s(\mathbf{P}\Delta_\mathrm{s}\mathbf{v})+2(\mu+\widetilde{\lambda})R^2\Delta_\mathrm{s}\,\mathrm{div}_\mathrm{s}\,\mathbf{v}-4(2\mu+\widetilde{\lambda})\frac{\mathsf{w}}{R}\Big)\\
&\quad + \Big(\frac{p}{2}+\frac{1}{6}\Big(\frac{h}{R}\Big)^3(\mu+2\widetilde{\lambda})\Big)\Big(R\Delta_\mathrm{s}\mathsf{w}+2\frac{\mathsf{w}}{R}\Big)\\
&\quad - \Big(p+2\frac{h}{R}(\mu+\tilde{\lambda})+\frac{1}{3}\Big(\frac{h}{R}\Big)^3\widetilde{\lambda}\Big)\Big(\mathrm{div}_\mathrm{s}\,\mathbf{v}+2\frac{\mathsf{w}}{R}\Big)-\zeta^2\rho_s hR\frac{\mathsf{w}}{R},\\
\mathbf{P}\boldsymbol{\Sigma}\boldsymbol{n} &= \Big(\frac{h}{R}\mu\Big(1+\frac{1}{3}\Big(\frac{h}{R}\Big)^2\Big)+\frac{p}{2}\Big)\Big(R\mathbf{P}\Delta_\mathrm{s}\mathbf{v}+2\frac{\mathbf{v}}{R}\Big)\\
&\quad + \frac{h}{R}(\mu+\tilde{\lambda})\Big(1+\frac{1}{3}\Big(\frac{h}{R}\Big)^2\Big)\nabla_\mathrm{s}(R\,\mathrm{div}_\mathrm{s}\,\mathbf{v}+2\mathsf{w})\\
&\quad - \frac{1}{6}\Big(\frac{h}{R}\Big)^3(2\mu+\widetilde{\lambda})\nabla_\mathrm{s}(R^2\Delta_\mathrm{s}\mathsf{w}+2\mathsf{w})-\zeta^2\rho_\mathrm{s}hR\frac{\mathbf{v}}{R},
\end{aligned}\tag{17}$$

where $\mathsf{w}=\mathbf{u}\cdot\boldsymbol{n}$ and $\mathbf{v}=\mathbf{P}\boldsymbol{u}$, are, respectively, the normal and the tangential components of $\mathbf{u}$.

Expansion in spherical harmonics.

To solve (16), we mimick the procedure of [3]. For $\boldsymbol{r}$ the position vector with respect to the center of B_R, we let

$$r=|\boldsymbol{r}|\qquad\text{and}\qquad\widehat{\boldsymbol{r}}=\boldsymbol{r}/r,\tag{18}$$

and we introduce the expansion

$$\mathsf{q}(\boldsymbol{r},\zeta)=\sum_{\ell\geq 0}\sum_{-\ell\leq m\leq\ell}Q_{\ell m}(r,\zeta)Y_{\ell m}(\widehat{\boldsymbol{r}})\tag{19}$$

of the pressure increment using spherical harmonics $\{Y_{\ell m}:\ell\geq 0,-\ell\leq m\leq\ell\}$. Spherical harmonics are a complete orthonormal set on the unit sphere. Using spherical polar coordinates (θ,ϕ), with $0\leq\theta\leq\pi$ the polar angle ($\theta=0$ on the North Pole) and $0\leq\phi<2\pi$ the azimuthal angle, spherical harmonics are defined by $Y_{\ell m}(\theta,\phi)=(-1)^m N_{\ell m}e^{im\phi}P_{\ell m}(\cos\theta)$, where $N_{\ell m}$ is a normalization constant and $P_{\ell m}:[-1,+1]\to\mathbb{R}$ is an associated Legendre polynomial. For $m\geq 0$, the associated Legendre polynomials are defined by $P_{\ell m}(x)=(-1)^m\left(1-x^2\right)^{m/2}\frac{d^m}{dx^m}\left(P_\ell(x)\right)$, with $P_\ell(x)$ the ℓ-th Legendre polynomial, defined by $P_0(x)=1$, $P_1(x)=x$, and by the recursion formula $(\ell+1)P_{\ell+1}(x)=(2\ell+1)xP_\ell(x)-\ell P_{\ell-1}(x)$. For $\ell\leq m<0$, $P_{\ell-m}(x)=(-1)^m\frac{(l-m)!}{(l+m)!}P_{\ell m}(x)$ [4].

The analogous of spherical harmonics for vector fields are the *vector spherical harmonics*,

defined on the unit sphere through [4, p. 1898]:

$$\begin{aligned} \mathbf{p}_{\ell m}(\widehat{\boldsymbol{r}}) &= Y_{\ell m}(\widehat{\boldsymbol{r}})\widehat{\mathbf{r}}, && \ell \geq 0, \\ \mathbf{b}_{\ell m}(\widehat{\boldsymbol{r}}) &= \frac{1}{s_\ell}\nabla_{\mathrm{s}} Y_{\ell m}(\widehat{\boldsymbol{r}}), && \ell \geq 0, \\ \mathbf{c}_{\ell m}(\widehat{\boldsymbol{r}}) &= \widehat{\boldsymbol{r}} \times \mathbf{b}_{\ell m}(\widehat{\boldsymbol{r}}), && \ell \geq 0, \end{aligned} \tag{20}$$

where ∇_{s} is the surface gradient on the unit sphere, and

$$s_\ell = \sqrt{\ell(\ell+1)}.$$

We remark that the first spherical harmonic $Y_{00}(\widehat{\boldsymbol{r}})$ is constant and, as a consequence,

$$\nabla Y_{00} = \mathbf{0}, \qquad \mathbf{b}_{00} = \mathbf{c}_{00} = \mathbf{0}. \tag{21}$$

Vector spherical harmonics are a complete orthonormal set on the unit sphere. Accordingly, the displacement $\mathbf{u}(\boldsymbol{r}, \zeta)$ admits the expansion:

$$\mathbf{u}(\boldsymbol{r}) = \sum_{\ell \geq 0} \sum_{-\ell \leq m \leq \ell} \mathbf{u}_{\ell m}(\boldsymbol{r}, \zeta), \tag{22}$$

with

$$\mathbf{u}_{\ell m}(\boldsymbol{r}, \zeta) = P_{\ell m}(r, \zeta)\mathbf{p}_{\ell m}(\widehat{\boldsymbol{r}}) + B_{\ell m}(r, \zeta)\mathbf{b}_{\ell m}(\widehat{\boldsymbol{r}}) + C_{\ell m}(r, \zeta)\mathbf{c}_{\ell m}(\widehat{\boldsymbol{r}}), \tag{23}$$

where, in view of (21), we have set

$$B_{00} = 0, \qquad C_{00} = 0. \tag{24}$$

Differential identities for spherical harmonics. With a view towards solving (16), we extend spherical harmonics and vector spherical harmonics to the entire space by letting $Y_{\ell m}(\boldsymbol{r}) = Y_{\ell m}(\widehat{\boldsymbol{r}})$, $\boldsymbol{p}_{\ell m}(\boldsymbol{r}) = \boldsymbol{p}_{\ell m}(\widehat{\boldsymbol{r}})$, etc. The resulting extensions satisfy [1]

$$\nabla Y_{\ell m}(\boldsymbol{r}) = \frac{s_\ell}{r}\mathbf{b}_{\ell m}(\boldsymbol{r}), \qquad -\Delta Y_{\ell m} = \frac{s_\ell^2}{r^2} Y_{\ell m}. \tag{25}$$

Moreover,

$$\mathrm{div}\mathbf{p}_{\ell m} = \frac{2}{r} Y_{\ell m}, \quad \mathrm{div}\mathbf{b}_{\ell m} = -\frac{s_\ell}{r} Y_{\ell m}, \quad \mathrm{div}\mathbf{c}_{\ell m} = 0, \tag{26}$$

and

$$\begin{aligned} \Delta \mathbf{p}_{\ell m} &= -\Big(\frac{s_\ell^2 + 2}{r^2}\Big)\mathbf{p}_{\ell m} + \frac{2 s_\ell}{r^2}\mathbf{b}_{\ell m}, \\ \Delta \mathbf{b}_{\ell m} &= -\frac{s_\ell^2}{r^2}\mathbf{b}_{\ell m} + \frac{2 s_\ell}{r^2}\mathbf{p}_{\ell m}, \\ \Delta \mathbf{c}_{\ell m} &= -\frac{s_\ell^2}{r^2}\mathbf{c}_{\ell m}. \end{aligned} \tag{27}$$

Solution in the vitreous

The identities (25)–(27) make the choice of spherical harmonics particularly useful to solve (16)–(17). In fact, by (25)–(27), we have

$$\begin{aligned}\Delta \mathbf{u}_{\ell m}(\boldsymbol{r},\zeta) = \quad & \Big(\Big(D_\ell - \frac{2}{r^2}\Big)P_{\ell m}(r,\zeta) + \frac{2s_\ell}{r}B_{\ell m}(r,\zeta)\Big)\mathbf{p}_{\ell m}(\boldsymbol{r}) \\ & + \Big(\frac{2s_\ell}{r^2}P_{\ell m}(r,\zeta) + D_\ell B_{\ell m}(r,\zeta)\Big)\mathbf{b}_{\ell m}(\boldsymbol{r}) \\ & + D_\ell C_{\ell m}(r,\zeta)\mathbf{c}_{\ell m}(\boldsymbol{r}),\end{aligned} \tag{28}$$

where

$$D_\ell = \frac{d^2}{dr^2} + \frac{2}{r}\frac{d}{dr} - \frac{s_\ell^2}{r^2}. \tag{29}$$

Furthermore, by making use of (26), we find

$$\operatorname{div}\mathbf{u}_{\ell m}(\boldsymbol{r},\zeta) = \Big(\frac{2}{r}P_{\ell m}(r,\zeta) - \frac{s_\ell}{r}B_{\ell m}(r,\zeta)\Big)Y_{\ell m}(\boldsymbol{r}). \tag{30}$$

Moreover, on setting $\mathsf{q}_{\ell m}(\boldsymbol{r},\zeta) = Q_{\ell m}(r,\zeta)Y_{\ell m}(\widehat{\boldsymbol{r}})$,

$$\nabla \mathsf{q}_{\ell m}(\boldsymbol{r},\zeta) = \frac{dQ_{\ell m}}{dr}(r,\zeta)\mathbf{p}_{\ell m}(\boldsymbol{r}) + Q_{\ell m}(r,\zeta)\frac{s_\ell}{r}\mathbf{b}_{\ell m}(\boldsymbol{r}). \tag{31}$$

We remark that, although Y_{00} is constant, the vector spherical harmonic $\mathbf{p}_{00}(\boldsymbol{r}) = Y_{00}\widehat{\boldsymbol{r}}$ is not.

Now, the key observation is that the action on the spherical harmonics of the operators Δ, div, and ∇, which appear in the motion equations (16), does not mix coefficients with different values of ℓ and m. Keeping this observation in mind, we substitute the expansions (19) and (22) into the governing equations (16) and we use (28) and (31), as well as the aforementioned orthogonality property to obtain a series of uncoupled systems, each for a particular value of $\ell \geq 0$ and $-\ell \leq m \leq \ell$.

The resulting systems have to be treated differently, according to whether $\ell = 0$ or $\ell \geq 1$. As we shall see below, the corresponding solutions are trivial in the first case.

Coefficients with $\boldsymbol{\ell}$=0. For $\ell = 0$ we obtain the following system for $P_{00}(r,\zeta)$ and $Q_{00}(r,\zeta)$:

$$\begin{aligned}&\rho_{\mathrm{v}}\zeta^2 P_{00} = -\frac{dQ_{00}}{dr} + \mathsf{G}(\zeta)\left(\frac{\mathrm{d}^2}{\mathrm{d}r^2} + \frac{2}{r}\frac{\mathrm{d}}{\mathrm{d}r} - \frac{2}{r^2}\right)P_{00}, \\ &0 = \frac{2}{r}P_{00} + \frac{dP_{00}}{dr}.\end{aligned} \tag{32}$$

All solutions of the second equation are proportional to $1/r^2$. Thus, the requirement that solutions are bounded demands that $P_{00}(r,\zeta) = 0$. Then, the first of (34) imposes that $Q_{00}(r,\zeta)$ be a constant. Thus,

$$Q_{00}(r,\zeta) = -C_{00}^{(1)}, \qquad P_{00}(r,\zeta) = 0, \tag{33}$$

with $C_{00}^{(1)}$ an integration constant. This solution describes the equilibrium configuration with an arbitrary pressure.

The case $\ell \geq 1$. For each $\ell \geq 1$ and $-\ell \leq m \leq \ell$, we multiply both sides of the first of (16) by, respectively, $\mathbf{p}_{\ell m}$, $\mathbf{b}_{\ell m}$ and $\mathbf{b}_{\ell m}$, and we multiply both sides of the second of (16) by $Y_{\ell m}$. Integrating on the unit sphere and making use of the aforementioned orthogonality properties, we obtain a system of four differential equations:

$$\begin{aligned}
&\rho_{\mathrm{v}}\zeta^2 P_{\ell m} = -\frac{dQ_{\ell m}}{dr} + \mathsf{G}(\zeta)\left(\left(D_\ell - \frac{2}{r^2}\right)P_{\ell m} + \frac{2s_\ell}{r^2}B_{\ell m}\right),\\
&\rho_{\mathrm{v}}\zeta^2 B_{\ell m} = -\frac{s_\ell}{r}Q_{\ell m} + \mathsf{G}(\zeta)\left(\frac{2s_\ell}{r^2}P_{\ell m} + D_\ell B_{\ell m}\right),\\
&0 = \frac{2}{r}P_{\ell m} + \frac{dP_{\ell m}}{dr} - \frac{s_\ell}{r}B_{\ell m},\\
&\rho_{\mathrm{v}}\zeta^2 C_{\ell m} = \mathsf{G}(\zeta)D_\ell C_{\ell m},
\end{aligned} \tag{34}$$

where we have set

$$D_\ell = \frac{d^2}{dr^2} + \frac{2}{r}\frac{d}{dr} - \frac{s_\ell^2}{r^2}. \tag{35}$$

The first three equations of (34) are uncoupled from the last one; their solution can be obtained by elimination of the unknowns $B_{\ell m}(r,\zeta)$ (using the third equation) and $Q_{\ell m}(r,\zeta)$ (using the second equation). This leads to the fourth-order differential equation for $P_{\ell m}(r,\zeta)$:

$$\begin{aligned}
&\frac{\mathsf{G}(\zeta)(-2+s_\ell^2)P_{\ell m}(r)}{r} + r\rho_v\zeta^2 P_{\ell m}(r) =\\
&\frac{1}{2s_\ell}\left(8Gs_\ell\frac{dP_{\ell m}}{dr} + \mathsf{G}(\zeta)r(-12\sqrt{2} + s_\ell(2+\sqrt{2}s_\ell))\frac{d^2P_{\ell m}}{dr^2}\right.\\
&\left. + \sqrt{2}r\left(\rho_v\zeta^2\left(2P_{\ell m}(r) + r\left(4\frac{dP_{\ell m}}{dr} + r\frac{d^2P_{\ell m}}{dr^2}\right)\right) - \mathsf{G}(\zeta)r\left(8\frac{d^3P_{\ell m}}{dr^3} + r\frac{d^4P_{\ell m}}{dr^4}\right)\right)\right).
\end{aligned} \tag{36}$$

The solution of (36) depends, in general, on four integration constants. These constants determine $B_{\ell m}(r,\zeta)$ and $Q_{\ell m}(r,\zeta)$, respectively through the second and the third of (34). Among these solutions, we retain as admissible those which are bounded. Likewise, the fourth equation of (34) determines $C_{\ell m}(r,\zeta)$ to within two integration constants. The result is:

$$\begin{aligned}
Q_{\ell m}(r,\zeta) &= -C^{(1)}_{\ell m}\frac{\rho_{\mathrm{v}}R\zeta^2}{\ell}\left(\frac{r}{R}\right)^{\ell},\\
P_{\ell m}(r,\zeta) &= C^{(1)}_{\ell m}\left(\frac{r}{R}\right)^{\ell-1} + C^{(2)}_{\ell m}\left(\frac{r}{R}\right)^{-1} j_\ell\left(a(\zeta)\frac{r}{R}\right),\\
B_{\ell m}(r,\zeta) &= C^{(1)}_{\ell m}\frac{s_\ell}{\ell}\left(\frac{r}{R}\right)^{\ell-1} + \frac{C^{(2)}_{\ell m}}{s_\ell}\left(a(\zeta)\, j_{\ell-1}\left(a(\zeta)\frac{r}{R}\right) - \ell\left(\frac{r}{R}\right)^{-1} j_\ell\left(a(\zeta)\frac{r}{R}\right)\right),\\
C_{\ell m}(r,\zeta) &= C^{(3)}_{\ell m} j_\ell\left(a(\zeta)\frac{r}{R}\right),
\end{aligned} \tag{37}$$

where $j_\ell(x) = \sqrt{\pi/(2x)}J_{\ell+1/2}(x)$ denotes the ℓ-th spherical Bessel function,

$$a(\zeta) = iR\zeta\sqrt{\rho_{\mathrm{v}}/\mathsf{G}(\zeta)}, \tag{38}$$

and $C^{(1)}_{\ell m}$, $C^{(2)}_{\ell m}$ and $C^{(3)}_{\ell m}$ are three constants.

Concerning the coefficients with $\ell = 1$, the smoothness of the displacement at the origin imposes (see [3] and references cited therein) that $B_{1m}(0) = \sqrt{2}P_{1m}(0)$ and $P'_{1m}(0) = B'_{1m}(0) = 0$. The first two spherical Bessel functions, for small values of the argument z, can be approximated as

$$j_0(z) = \frac{\sin z}{z} = 1 + o(z), \qquad j_1(z) = \frac{\sin z}{z^2} - \frac{\cos z}{z} = \frac{1}{2}z + o(z^2). \tag{39}$$

Thus, near $r = 0$,

$$\begin{aligned} P_{1m}(r,\zeta) &= C^{(1)}_{1m} + \frac{1}{2}C^{(2)}_{1m}a(\zeta) + o(r), \\ B_{1m}(r,\zeta) &= \sqrt{2}C^{(1)}_{1m} + \frac{1}{2}\frac{C^{(2)}_{1m}}{\sqrt{2}}a(\zeta) + o(r). \end{aligned} \tag{40}$$

Thus $P'_{1m}(0) = B'_{1m}(0) = 0$ is verified for all choices of $C^{(1)}_{1m}$ and $C^{(2)}_{1m}$. On the other hand, the requirement that $B_{1m}(0) = \sqrt{2}P_{1m}(0)$ yields the condition

$$C^{(2)}_{1m} = 0 \tag{41}$$

As a result, we have

$$\begin{aligned} P_{1m}(r,\zeta) &= C^{(1)}_{1m} \\ B_{1m}(r,\zeta) &= \sqrt{2}C^{(1)}_{1m}, \end{aligned} \tag{42}$$

and using the properties of the spherical harmonics Y_{1m} one can check that the resulting displacement is uniform (i.e., a translation). As one can guess, the only oscillatory motion compatible with a uniform translation is the trivial one, and this shall be confirmed in the analysis carried out in the next section.

Matching conditions.

The integration constants $C^{(i)}_{\ell m}$ are determined by enforcing the matching conditions (17). To convert (17) into conditions at $r = R$ on the functions $Q_{\ell m}(r,\zeta)$, $P_{\ell m}(r,\zeta)$, $B_{\ell m}(r,\zeta)$, and $C_{\ell m}(r,\zeta)$, we proceed in two steps. In the first step, we use (19) and (22) to compute the left-hand sides of the matching conditions (16). The calculation is quite straightforward, and leads to:

$$\boldsymbol{n}\cdot\boldsymbol{\Sigma}\boldsymbol{n} + p\nabla w\cdot\boldsymbol{n} = \sum_{\ell\geq 0}\sum_{-\ell\leq m\leq\ell}\Big(-Q_{\ell m}(R,\zeta) + (2\mathsf{G}(\zeta)+p)\frac{dP_{\ell m}}{dr}(R,\zeta)\Big)Y_{\ell m}(\widehat{\boldsymbol{r}}), \tag{43}$$

and

$$\begin{aligned} \mathbf{P}\boldsymbol{\Sigma}\boldsymbol{n} =& \mathsf{G}(\zeta)\sum_{\ell\geq 0}\sum_{-\ell\leq m\leq\ell}\bigg(\Big(\frac{dB_{\ell m}}{dr}(R,\zeta) - \frac{B_{\ell m}(R,\zeta)}{R} + \frac{s_\ell}{R}P_{\ell m}(R,\zeta)\Big)\mathbf{b}_{\ell m}(\widehat{\boldsymbol{r}}) \\ &+ \Big(\frac{dC_{\ell m}}{dr}(R,\zeta) - \frac{C_{\ell m}(R,\zeta)}{R}\Big)\mathbf{c}_{\ell m}(\widehat{\boldsymbol{r}})\bigg), \end{aligned} \tag{44}$$

where we have set $\mathbf{b}_{0m} = \mathbf{0}$ and $\mathbf{c}_{0m} = \mathbf{0}$.

In the second step, we substitute the expansions (22) into the right-hand sides of the matching conditions (16). This step is a bit more elaborate than the previous one, because there are numerous terms to compute. To make the calculation more organized, we write the tangential and normal components of the displacement on S_R as

$$\mathsf{v}(\boldsymbol{r},\zeta) = \sum_{\ell\geq 1}\sum_{-\ell\leq m\leq \ell} \mathsf{v}_{\ell m}(\boldsymbol{r},\zeta), \qquad \mathsf{w}(\boldsymbol{r},\zeta) = \sum_{\ell\geq 0}\sum_{-\ell\leq m\leq \ell} \mathsf{w}_{\ell m}(\boldsymbol{r},\zeta) \tag{45}$$

where

$$\mathsf{v}_{\ell m}(\boldsymbol{r}) = B_{\ell m}(R,\zeta)\mathbf{b}_{\ell m}(\boldsymbol{r}) + C_{\ell m}(R,\zeta)\mathbf{c}_{\ell m}(\boldsymbol{r}), \tag{46}$$

and

$$\mathsf{w}_{\ell m}(\boldsymbol{r},\zeta) = P_{\ell m}(R,\zeta)Y_{\ell m}(\boldsymbol{r}). \tag{47}$$

Then, we compute the action on v and w of the linear operators appearing on the right-hand sides of (16) by superposition on each term of the summations in (45):

$$\begin{aligned}
&\nabla_{\mathrm{s}}\mathsf{w}_{\ell m} = \frac{s_\ell}{R}P_{\ell m}\mathbf{b}_{\ell m} && \Delta_{\mathrm{s}}\mathsf{w}_{\ell m} = -\frac{s_\ell^2}{R^2}P_{\ell m}Y_{\ell m},\\
&\nabla_{\mathrm{s}}\Delta_{\mathrm{s}}\mathsf{w}_{\ell m} = -\frac{s_\ell^3}{R^3}P_{\ell m}\mathbf{b}_{\ell m}, && \Delta_{\mathrm{s}}\Delta_{\mathrm{s}}\mathsf{w}_{\ell m} = \frac{s_\ell^4}{R^4}P_{\ell m}Y_{\ell m}\\
&\mathrm{div}_{\mathrm{s}}\mathsf{v}_{\ell m} = -\frac{s_\ell}{R}B_{\ell m}Y_{\ell m}, && \\
&\mathbf{P}\Delta_{\mathrm{s}}\mathsf{v}_{\ell m} = -\frac{s_\ell^2}{R^2}B_{\ell m}\mathbf{b}_{\ell m} - \frac{s_\ell^2}{R^2}C_{\ell m}\mathbf{c}_{\ell m} && \nabla_{\mathrm{s}}\mathrm{div}_{\mathrm{s}}\mathsf{v}_{\ell m} = -\frac{s_\ell^2}{R^2}B_{\ell m}\mathbf{b}_{\ell m},\\
&\Delta_{\mathrm{s}}\mathrm{div}_{\mathrm{s}}\mathsf{v}_{\ell m} = \frac{s_\ell^3}{R^3}B_{\ell m}Y_{\ell m}, && \mathrm{div}_{\mathrm{s}}\mathbf{P}\Delta_{\mathrm{s}}\mathsf{v}_{\ell m} = \frac{s_\ell^3}{R^3}B_{\ell m}Y_{\ell m},
\end{aligned} \tag{48}$$

To deduce (48), it suffices to notice that the identities (25)–(27) hold also when the differential operators ∇, div and Δ are replaced by the surface differential operators ∇_{s}, $\mathrm{div}_{\mathrm{s}}$ and Δ_{s} acting on the restrictions of their argument on the sphere S_R. The calculations are routine, but for completeness, we prefer to work them out.

Since $Y_{\ell m}$ does not depend on the radial distance r from the origin, on S_R we have $\nabla Y_{\ell m}\cdot\boldsymbol{n} = 0$, and therefore,

$$\nabla Y_{\ell m} = \mathbf{P}\nabla Y_{\ell m} =: \nabla_{\mathrm{s}}Y_{\ell m}.$$

Moreover, on denoting by ∂_r the derivative in the radial direction, we have, on S_R,

$$\nabla\nabla Y_{\ell m} = \nabla\nabla_{\mathrm{s}}Y_{\ell m} = \nabla_{\mathrm{s}}\nabla_{\mathrm{s}}Y_{\ell m} + \partial_r(\nabla_{\mathrm{s}}Y_{\ell m})\otimes\boldsymbol{n}.$$

Since $\nabla_{\mathrm{s}}Y_{\ell m}$ is orthogonal to $\boldsymbol{n}$, we have $\partial_r\nabla_{\mathrm{s}}Y_{\ell m}\cdot\boldsymbol{n} = 0$, thus (recall that : is the contraction between tensors):

$$\begin{aligned}
\Delta Y_{\ell m} &:= \mathrm{div}\nabla Y_{\ell m} = \mathrm{tr}(\nabla\nabla Y_{\ell m}) = \mathrm{tr}(\nabla_{\mathrm{s}}\nabla_{\mathrm{s}}Y_{\ell m}) + \partial_r\nabla_{\mathrm{s}}Y_{\ell m}\cdot\boldsymbol{n}\\
&= \mathrm{div}_{\mathrm{s}}\nabla_{\mathrm{s}}Y_{\ell m} = \Delta_{\mathrm{s}}Y_{\ell m}.
\end{aligned}$$

In a similar fashion, since $\partial_r \mathbf{p}_{\ell m} = 0$,

$$\mathrm{div}\mathbf{p}_{\ell m} = \mathrm{tr}(\nabla \mathbf{p}_{\ell m}) = \mathrm{tr}(\nabla_{\mathrm{s}} \mathbf{p}_{\ell m}) =: \mathrm{div}_{\mathrm{s}} \mathbf{p}_{\ell m}.$$

It is also easy to check that $\mathrm{div}\mathbf{b}_{\ell m} = \mathrm{div}_{\mathrm{s}}\mathbf{b}_{\ell m}$, $\mathrm{div}\mathbf{c}_{\ell m} = \mathrm{div}_{\mathrm{s}}\mathbf{c}_{\ell m} = 0$. To show that $\Delta\mathbf{p}_{\ell m} = \Delta_{\mathrm{s}}\mathbf{p}_{\ell m}$, we take a constant vector $\boldsymbol{a}$ and we apply the definition of divergence of a tensor field to $\nabla\mathbf{p}_{\ell m}$:

$$\boldsymbol{a} \cdot \Delta\mathbf{p}_{\ell m} = \boldsymbol{a} \cdot \mathrm{div}\nabla\mathbf{p}_{\ell m} = \mathrm{div}(\nabla\mathbf{p}_{\ell m}^{\top}\boldsymbol{a}) = \mathrm{div}\nabla(\mathbf{p}_{\ell m} \cdot \boldsymbol{a}) = \mathrm{div}\nabla_{\mathrm{s}}(\mathbf{p}_{\ell m} \cdot \mathbf{a}), \tag{49}$$

where we use the fact that $\mathbf{b}_{\ell m}$ does not depend on r. Moreover,

$$\begin{aligned}\mathrm{div}\nabla_{\mathrm{s}}(\mathbf{p}_{\ell m} \cdot \boldsymbol{a}) &= \mathrm{tr}(\nabla\nabla_{\mathrm{s}}(\mathbf{p}_{\ell m} \cdot \boldsymbol{a})) = \mathrm{tr}(\nabla_{\mathrm{s}}\nabla_{\mathrm{s}}(\mathbf{p}_{\ell m} \cdot \mathbf{a})) + \mathrm{tr}(\partial_r\nabla_{\mathrm{s}}(\mathbf{p}_{\ell m} \cdot \boldsymbol{a}) \otimes \widehat{\boldsymbol{r}}) \\ &= \mathrm{tr}(\nabla_{\mathrm{s}}\nabla_{\mathrm{s}}(\mathbf{p}_{\ell m} \cdot \mathbf{a})) = \mathrm{div}_{\mathrm{s}}\nabla_{\mathrm{s}}(\mathbf{p}_{\ell m} \cdot \boldsymbol{a}) = \mathrm{div}_{\mathrm{s}}(\nabla_{\mathrm{s}}\mathbf{p}_{\ell m}^{\top}\boldsymbol{a}) = \boldsymbol{a} \cdot \mathrm{div}_{\mathrm{s}}\nabla_{\mathrm{s}}\mathbf{p}_{\ell m} \\ &= \boldsymbol{a} \cdot \Delta_{\mathrm{s}}\mathbf{p}_{\ell m}.\end{aligned} \tag{50}$$

Analogous calculations hold for $\mathbf{b}_{\ell m}$ and $\mathbf{c}_{\ell m}$. With the above observation, and resorting to the identities (25)–(27) we can compute the action on spherical harmonics of the operators appearing on the right-hand sides of the boundary conditions (17):

$$\begin{aligned} &\nabla_{\mathrm{s}}\Delta_{\mathrm{s}}Y_{\ell m} = -\frac{s_\ell^3}{R^3}\mathbf{b}_{\ell m}, && \Delta_{\mathrm{s}}\Delta_{\mathrm{s}}Y_{\ell m} = \frac{s_\ell^4}{R^4}Y_{\ell m} && \\ &\nabla_{\mathrm{s}}\mathrm{div}_{\mathrm{s}}\mathbf{p}_{\ell m} = \frac{2s_\ell}{R^2}\mathbf{b}_{\ell m}, && \nabla_{\mathrm{s}}\mathrm{div}_{\mathrm{s}}\mathbf{b}_{\ell m} = -\frac{s_\ell^2}{R^2}\mathbf{b}_{\ell m}, && \nabla_{\mathrm{s}}\mathrm{div}_{\mathrm{s}}\mathbf{c}_{\ell m} = \mathbf{0}, \\ &\mathrm{div}_{\mathrm{s}}\mathbf{P}\Delta_{\mathrm{s}}\mathbf{p}_{\ell m} = -\frac{4s_\ell}{R^3}Y_{\ell m}, && \mathrm{div}_{\mathrm{s}}\mathbf{P}\Delta_{\mathrm{s}}\mathbf{b}_{\ell m} = \frac{s_\ell^3}{R^3}Y_{\ell m}, && \mathrm{div}_{\mathrm{s}}\mathbf{P}\Delta_{\mathrm{s}}\mathbf{c}_{\ell m} = \mathbf{0} \\ &\Delta_{\mathrm{s}}\mathrm{div}_{\mathrm{s}}\mathbf{p}_{\ell m} = -\frac{2s_\ell^2}{R^3}Y_{\ell m}, && \Delta_{\mathrm{s}}\mathrm{div}_{\mathrm{s}}\mathbf{b}_{\ell m} = \frac{s_\ell^3}{R^3}Y_{\ell m}, && \Delta_{\mathrm{s}}\mathrm{div}_{\mathrm{s}}\mathbf{c}_{\ell m} = \mathbf{0} \end{aligned} \tag{51}$$

Substituting (45) into the right-hand sides of (17) we obtain

$$\begin{aligned}\boldsymbol{n} \cdot \boldsymbol{\Sigma}\boldsymbol{n} = \sum_{\ell \geq 0} \sum_{-\ell \leq m \leq \ell} \Bigg\{ \Bigg[&- \left(\left(\frac{s_\ell^2}{2} - 1 \right) p + 2p + 4\frac{h}{R}(\mu + \widetilde{\lambda}) + \zeta^2 \rho_{\mathrm{s}} h R \right) \\ &+ \frac{1}{12}\left(\frac{h}{R}\right)^3 \Big(-(4 + s_\ell^4)(2\mu + \widetilde{\lambda}) + 2(2 - s_\ell^2)(\mu + 2\widetilde{\lambda}) - 8\widetilde{\lambda} \Big) \Bigg] \frac{P_{\ell m}(R, \zeta)}{R} \\ &+ \left[s_\ell \Big(p + 2\frac{h}{R}(\mu + \widetilde{\lambda}) \Big) + \frac{1}{12}\left(\frac{h}{R}\right)^3 \Big(4\widetilde{\lambda}s_\ell + 2(2\mu + \widetilde{\lambda})s_\ell^3 \Big) \right] \frac{B_{\ell m}(R, \zeta)}{R} \Bigg\} Y_{\ell m}(\boldsymbol{r}),\end{aligned} \tag{52}$$

and

$$\mathbf{P}\boldsymbol{\Sigma}\boldsymbol{n} = \sum_{\ell\geq 0}\sum_{-\ell\leq m\leq\ell}\Bigg\{\Big[2s_\ell\frac{h}{R}(\mu+\widetilde{\lambda}) + \frac{1}{6}\Big(\frac{h}{R}\Big)^3 s_\ell\Big(4(\mu+\widetilde{\lambda}) - (2-s_\ell^2)(2\mu+\widetilde{\lambda})\Big)\Big]\frac{P_{\ell m}(R,\zeta)}{R}$$
$$+\Big[(2-s_\ell^2)\Big(\frac{h}{R}\mu+\frac{p}{2}\Big) - s_\ell^2\frac{h}{R}(\mu+\widetilde{\lambda}) - \zeta^2\rho_{\mathrm{s}}hR + \frac{1}{3}\Big(\frac{h}{R}\Big)^3((2-s_\ell^2)\mu - s_\ell^2(\mu+\widetilde{\lambda}))\Big]\frac{B_{\ell m}(R,\zeta)}{R}\Bigg\}\mathbf{b}_{\ell m}(\boldsymbol{r})$$
$$+\Big[(2-s_\ell^2)\Big(\frac{h}{R}\mu+\frac{p}{2}+\frac{1}{3}\Big(\frac{h}{R}\Big)^3\mu\Big) - \zeta^2\rho_{\mathrm{s}}hR\Big]\frac{C_{\ell m}(R,\zeta)}{R}\mathbf{c}_{\ell m}(\boldsymbol{r}). \tag{53}$$

Here, again, we have adopted the convention that $\mathbf{b}_{0m} = \mathbf{0}$ and $\mathbf{c}_{0m} = \mathbf{0}$.

Equating the right-hand sides of (43)–(44) with the right–hand sides of (52)–(53) and using the orthogonality properties of spherical and vector spherical harmonics we obtain, for $\ell = 0$, the equation

$$A_{11}(0)\frac{P_{00}(R,\zeta)}{R} + Q_{00}(R,\zeta) = 0, \tag{54}$$

and, for $\ell \geq 1$, the following system:

$$\begin{aligned}
&A_{11}(\ell,\zeta)\frac{P_{\ell m}(R,\zeta)}{R} + A_{12}(\ell)\frac{B_{\ell m}(R,\zeta)}{R} + D_{11}(\zeta)\frac{dP_{\ell m}}{dr}(R,\zeta) + Q_{\ell m}(R,\zeta) = 0,\\
&A_{21}(\ell,\zeta)\frac{P_{\ell m}(R,\zeta)}{R} + A_{22}(\ell,\zeta)\frac{B_{\ell m}(R,\zeta)}{R} + D_{22}(\zeta)\frac{dB_{\ell m}}{dr}(R,\zeta) = 0,\\
&A_{33}(\ell,\zeta)\frac{C_{\ell m}(R,\zeta)}{R} + D_{33}(\zeta)\frac{dC_{\ell m}}{dr}(R,\zeta) = 0,
\end{aligned} \tag{55}$$

where the coefficients are given by

$$\begin{aligned}
A_{11}(\ell,\zeta) &= -Rh\rho_{\mathrm{s}}\zeta^2 - \left(\left(\frac{s_\ell^2}{2}-1\right)p + 2p + 4\frac{h}{R}(\mu+\widetilde{\lambda})\right)\\
&\quad - \frac{1}{12}\left(\frac{h}{R}\right)^3\left(2(2+s_\ell^2+s_\ell^4)\mu + (2+s_\ell^2)^2\widetilde{\lambda}\right),\\
A_{12}(\ell) &= s_\ell\Big(p + 2\frac{h}{R}(\mu+\widetilde{\lambda})\Big) + \frac{s_\ell}{6}\Big(\frac{h}{R}\Big)^3\Big(2s_\ell^2\mu + (2+s_\ell^2)\widetilde{\lambda}\Big),\\
A_{21}(\ell,\zeta) &= -s_\ell\mathsf{G}(\zeta) + A_{12}(\ell)\\
A_{22}(\ell,\zeta) &= -Rh\rho_{\mathrm{s}}\zeta^2 + \mathsf{G}(\zeta) + (2-s_\ell^2)\Big(\frac{h}{R}\mu+\frac{p}{2}\Big) - s_\ell^2\frac{h}{R}(\mu+\widetilde{\lambda})\\
&\quad + \frac{1}{3}\Big(\frac{h}{R}\Big)^3\Big(2(1-s_\ell^2)\mu - s_\ell^2\widetilde{\lambda}\Big),\\
A_{33}(\ell,\zeta) &= \mathsf{G}(\zeta) - Rh\rho_{\mathrm{s}}\zeta^2 + \big(2-s_\ell^2\big)\left(\frac{h}{R}\mu + \frac{p}{2} + \frac{1}{3}\Big(\frac{h}{R}\Big)^3\mu\right),\\
D_{11}(\zeta) &= -2\mathsf{G}(\zeta) + p,\\
D_{22}(\zeta) &= D_{33}(\zeta) = -\mathsf{G}(\zeta).
\end{aligned} \tag{56}$$

Note that the left-hand sides of (55) have physical dimensions of pressure/time.

Characteristic equation

We now substitue into (54) and (55) the representation formulas (33) and (37). Again, we must treat separately the cases $\ell = 0$, and $\ell \geq 1$. The case $\ell = 0$ is however trivial. In fact, in view of (33), (54) yields $C_{00}^{(1)} = 0$. Thus we conclude that $P_{00}(r,\zeta) = 0$ and $Q_{00}(r,\zeta) = 0$.

For $\ell \geq 1$ we substitute the representation (37) into (55), and we use the recurrence properties of the spherical Bessel functions to the homogeneous system

$$\begin{pmatrix} M_{11}(\ell,\zeta) & M_{11}(\ell,\zeta) \\ M_{11}(\ell,\zeta) & M_{11}(\ell,\zeta) \end{pmatrix} \begin{pmatrix} C_{\ell m}^{(1)} \\ C_{\ell m}^{(2)} \end{pmatrix} = \mathbf{0}, \tag{57}$$

where

$$\begin{aligned} M_{11}(\ell,\zeta) &= \frac{1}{R}\left[A_{11}(\ell,\zeta) + A_{12}(\ell)\frac{s_\ell}{\ell} + D_{11}(\zeta)(\ell-1)\right] - \frac{\zeta^2}{\ell}R\rho_{\mathrm{v}}, \\ M_{12}(\ell,\zeta) &= \frac{1}{R}\Bigg[A_{11}(\ell,\zeta)j_\ell(a(\zeta)) + A_{12}(\ell)\left(\frac{a(\zeta)}{s_\ell}j_{\ell-1}(a(\zeta)) - \frac{\ell}{s_\ell}j_\ell(a(\zeta))\right) \\ &\qquad + D_{11}(\zeta)\left(-(\ell+2)j_\ell(a(\zeta)) + a(\zeta)j_{\ell-1}(a(\zeta))\right)\Bigg], \\ M_{21}(\ell,\zeta) &= \frac{1}{R}\left[A_{21}(\ell,\zeta) + A_{22}(\ell,\zeta)\frac{s_\ell}{\ell} + D_{22}(\zeta)\frac{s_\ell}{\ell}(\ell-1)\right], \\ M_{22}(\ell,\zeta) &= \frac{1}{R}\Bigg[A_{21}(\ell,\zeta)j_\ell(a(\zeta)) + A_{22}\left(\frac{a(\zeta)}{s_\ell}j_{\ell-1}(a(\zeta)) - \frac{\ell}{s_\ell}j_\ell(a(\zeta))\right) \\ &\qquad + D_{22}\left(-\frac{a(\zeta)}{s_\ell}\left(j_{\ell-1}(a(\zeta)) + a(\zeta)j_\ell(a(\zeta))\right) + \frac{\ell}{s_\ell}(\ell+2)j_\ell(a(\zeta))\right)\Bigg], \end{aligned} \tag{58}$$

and the homogeneous equation

$$M_{33}(\ell,\zeta)C_{\ell m}^{(3)} = 0, \tag{59}$$

where

$$M_{33}(\ell,\zeta) = \frac{1}{R}\left[A_{33}(\ell,\zeta)j_\ell(a(\zeta)) + D_{33}(\zeta)\left(-(\ell+1)j_\ell(a(\zeta)) + a(\zeta)j_{\ell-1}(a(\zeta))\right)\right]. \tag{60}$$

For $\ell = 1$, the system (57) is supplemented by the regularity condition (41), which we rewrite here for the reader's convenience:

$$C_{1m}^{(2)} = 0. \tag{61}$$

Clearly, (57) and (61) constitute an overdetermined system, which admits only the trivial solutions, consistent with the expectation that during an oscillatory process the motion of the system cannot be a uniform displacement. Thus, the equation

$$M_{11}(\ell,\zeta)M_{22}(\ell,\zeta) - M_{12}(\ell,\zeta)M_{21}(\ell,\zeta) = 0, \tag{62}$$

for $\ell \geq 2$, and the equation

$$M_{33}(\ell,\zeta) = 0, \tag{63}$$

for $\ell \geq 1$ select the values of ζ for which oscillatory motions are possible.

Summing up, we obtain two family of modes. The modes of the first family, for $\ell \geq 2$, are a linear combination of the vector spherical harmonics $\mathbf{p}_{\ell m}$ and $\mathbf{b}_{\ell m}$, which possess both a radial and a tangential component. Modes of the second family, for $\ell \geq 1$, are proportional to the vector spherical harmonics $\mathbf{c}_{\ell m}$, which are purely tangential. We observe that the mode of the second family corresponding $\ell = 1$ does not entail a deformation of the shell.

The case of a pressurized spherical shell. The numerical solution of (63) and (62) involves a root-finding algorithm that requires an initial guess. To obtain this guess, we use the vibration frequencies from the uncoupled problem where a pressurized spherical shell vibrates freely. To obtain the equations that give these frequencies, we set to zero the right-hand sides of (17), which account for the effect of the material filling the shell. In terms of spherical harmonics, this is equivalent to setting to null the right-hand sides of (52) and (53). Then, on projecting these equations on each spherical harmonic, we obtain the following homogeneous system:

$$\begin{aligned} &A_{11}(\ell,\zeta)\frac{P_{\ell m}(R,\zeta)}{R} + A_{12}(\ell)\frac{B_{\ell m}(R,\zeta)}{R} = 0,\\ &A_{12}(\ell)\frac{P_{\ell m}(R,\zeta)}{R} + \overline{A}_{22}(\ell,\zeta)\frac{B_{\ell m}(R,\zeta)}{R} = 0,\\ &\overline{A}_{33}(\ell,\zeta)\frac{C_{\ell m}(R,\zeta)}{R} = 0, \end{aligned} \tag{64}$$

where $\overline{A}_{22}(\ell,\zeta) = A_{22}(\ell,\zeta) - \mathsf{G}(\zeta)$ and $\overline{A}_{33}(\ell,\zeta) = A_{33}(\ell,\zeta) - \mathsf{G}(\zeta)$. We note that (64) can be obtained from (55)–(56) by setting $\mathsf{G}(\zeta) = 0$, and by removing the unknown $Q_{\ell m}$. It's important to note that the volume enclosed by the shell may vary, as the incompressibility constraint within the shell is no longer present. This allows for an additional mode, characterized by an oscillatory radial movement, which, however, is not within the scope of our current study.

The characteristic equations are

$$A_{11}(\ell,\zeta)A_{22}(\zeta,\ell) - A_{12}^2(\ell) = 0, \qquad \overline{A}_{33}(\zeta,\ell) = 0. \tag{65}$$

The first is a quadratic equation with respect to the variable ζ^2. Writing the explicit form of the first equation would require too much space, and is not particularly informative. However, it is useful to note that for $\ell = 1$, the first equation reduces to

$$\zeta^2\Big(\zeta^2 + 2\Big(3 + \Big(\frac{h}{R}\Big)^2\Big)\frac{\lambda+\mu}{R^2\rho_{\mathrm{s}}}\Big) = 0, \tag{66}$$

which admits a null root. The eigenvectors of the matrix

$$\widetilde{M}(0) = \begin{pmatrix} A_{11}(1,0) & A_{12}(1) \\ A_{12}(1) & A_{22}(1,0) \end{pmatrix} \tag{67}$$

are proportional to $(1, \sqrt{2})$. The roots of the second equation in (65) are:

$$\zeta = \pm i \sqrt{\frac{s_\ell^2 - 2}{Rh\rho_s}\Big(\frac{h}{R}\mu + \frac{p}{2} + \frac{1}{3}\Big(\frac{h}{R}\Big)^3 \mu\Big)}. \tag{68}$$

It is immediate that they vanish for $\ell = 1$, since in that case $s_\ell^2 = 2$. The corresponding solution describes rigid rotations with constant angular velocity.

References

[1] R.G. Barrera, G.A. Estevez, and J. Giraldo. Vector spherical harmonics and their application to magnetostatics. *Eur. J. Phys.*, 6(4):287–294, 1985.

[2] M. Destrade, R. W. Ogden, and G. Saccomandi. Small amplitude waves and stability for a pre-stressed viscoelastic solid. *Z. angew. Math. Phys.*, 60:511–528, 2009.

[3] J. Meskauskas, R. Repetto, and J. H. Siggers. Oscillatory motion of a viscoelastic fluid within a spherical cavity. *J. Fluid Mech.*, 685:1–22, 2011.

[4] P. M. Morse and H. Feshbach. *Methods of theoretical physics.* McGraw-Hill, Boston, Mass, 1953.

[5] G. Tomassetti. A direct, coordinate-free approach to the mechanics of thin shells. ArXiV preprint: 2305.08884. 2023.
