# Supplementary material for: A mechanical model of ocular bulb vibrations and implications for acoustic tonometry
Source: PLoS One. 2024 Jan 18;19(1):e0294825. doi: 10.1371/journal.pone.0294825 (PMC10796012; doi:10.1371/journal.pone.0294825)
Supplement: Attachment — Submitted filename: response-to-reviewers.pdf [file pone.0294825.s002.pdf]

Response to Referee #1

We thank the referee for the time and effort spent reviewing our work. Please see the response to the referee's comment below.

- *The abstract and conclusions is clear but lacks quantitative data to support the statements regarding outcomes of the study, this should be addressed, especially in the conclusion.*

  We agree with the reviewer that the original version of the abstract failed to highlight the main results of the work. We have modified the abstract, trying to enphasize the main quantitative findings of the work.

  With the same aim, we have also added a final paragraph in the "Conclusions and Future Work" section.

- *The introduction section needs to add more information on the background and problem statement. Not much has been said about the importance of this study, and this is the weakness of the article.*

  We have added some background material in the "Introduction" (lines 32, 36, and 48). We also added a sentence concerning the problem statement on line 91). Despite these changes, we felt that the section where we had to work on more to highlight the importance of this study was "Conclusions and Future Work".

- *Methods were largely rigorous, and appropriate. They were also explained thoroughly.*

  We thank the reviewer for this positive comment.

- *The "Conclusions and Future Work" section appears excessively concise, then the authors should expand it by recapping all the steps to the proposed work, as to offer a brief but complete summary of it to the readers.*

  We have expanded the "Conclusions and Future Work". The paragraph on line 419 recaps the motivations of the work. The paragraph on line 473 summarizes our results. The last paragraph of the section is a discussion about our views of the potential of acoustic tonometry compared to other non-contact air puff tonometry techniques.

- *How can this research be used in certain clinical applications? Please discuss certain applications or give certain examples.*

  This point is now better addressed at the end of the "Conclusions and Future Work" section. In particular, we explain why acoustic tonometry should be preferable to the standard air puff tonometry.

Response to Referee #2

We thank the referee for the time and effort spent reviewing our work. Please see the response to the referee's comment below.

1. *What method was used to solve algebraic eigenvalue problem to determine natural frequencies of vibrations? Why this one was chosen from the many existing methods?*

   We have now specified that the nonlinear characteristic equations are solved numerically using the MATLAB (MathWorks®) function `fsolve`. As default, this function uses Powell's dog leg algorithm, which has proven to be robust and efficient in this case..

2. *There is no information about how numerical results were obtained. Which software packages were used?*

   See the reply to the previous point.

3. *It is necessary to check and put in order the list of references because the following articles are duplicated: No. 19 and No. 30, No. 20 and No. 32, No. 21 and No. 36.*

   We thank the reviewer for spotting the problems with the bibliography, which we have now fixed.

4. *Not all quantities and variables used in equations are explained in the text. For example, the parameter V in formula (5) is not defined in the article.*

   We have now carefully checked that all variables are introduced in the text (indeed, a few were not) and have amended the manuscript accordingly.

5. *On page 8 in line 254 (in the paragraph before eq. (25)) there is a reference to the equation of motion, but the equation number is indicated as (??).*

   We thank the reviewer for noting this. The reference has been corrected.

Response to Referee #3

We thank the referee for the time and effort spent reviewing our work. Please see the response to the referee's comment below.

It would be good to see the authors' opinion on the following issue: the results of all known tonometry methods, except for intraocular pressure measurement using the Ocular Response analyzer (ORA), are influenced by the biomechanical properties of eye shells. Are there any advantages in this regard in acoustic tonometry, which can be based on the proposed model? As shown in the work, vibration frequencies and damping rates are affected by both IOP and the mechanical properties of the corneoscleral shells. Then what is the advantage of acoustic tonometry other than non-contact measurement mode?

We have added a discussion regarding the point raised by the reviewer in the "Discussion and conclusions" section (last paragraph).
